# What is the Optimal Dipole Moment for Nonpolarizable Models of Liquids?

**DOI:** 10.1021/acs.jctc.2c01123

**Published:** 2023-02-24

**Authors:** Miguel Jorge, Maria Cecilia Barrera, Andrew W. Milne, Chris Ringrose, Daniel J. Cole

**Affiliations:** † Department of Chemical and Process Engineering, University of Strathclyde, 75 Montrose Street, Glasgow G1 1XJ, United Kingdom; ‡ Strathclyde Institute of Pharmacy and Biomedical Sciences, University of Strathclyde, 161 Cathedral St, Glasgow G4 0RE, United Kingdom; § School of Natural and Environmental Sciences, Newcastle University, Newcastle upon Tyne NE1 7RU, United Kingdom

## Abstract

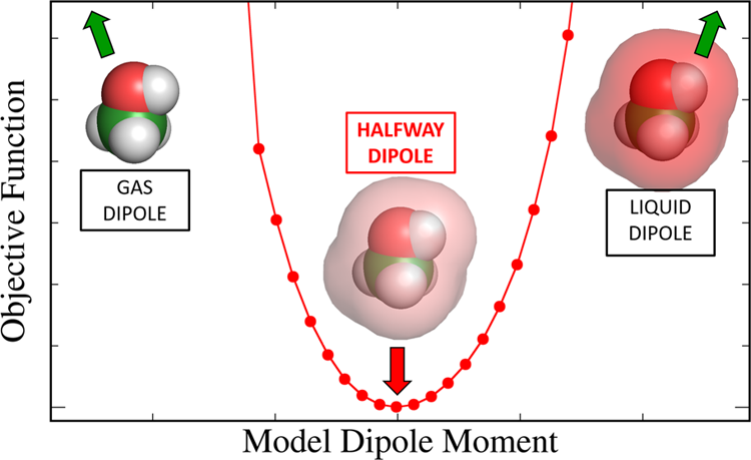

In classical nonpolarizable models, electrostatic interactions are usually described by assigning fixed partial charges to interaction sites. Despite the multitude of methods and theories proposed over the years for partial charge assignment, a fundamental question remains—what is the correct degree of polarization that a fixed-charge model should possess to provide the best balance of interactions (including induction effects) and yield the best description of the potential energy surface of a liquid phase? We address this question by approaching it from two separate and independent viewpoints: the QUantum mechanical BEspoke (QUBE) approach, which assigns bespoke force field parameters for individual molecules from *ab initio* calculations with minimal empirical fitting, and the Polarization-Consistent Approach (PolCA) force field, based on empirical fitting of force field parameters with an emphasis on transferability by rigorously accounting for polarization effects in the parameterization process. We show that the two approaches yield consistent answers to the above question, namely, that the dipole moment of the model should be approximately halfway between those of the gas and the liquid phase. Crucially, however, the reference liquid-phase dipole needs to be estimated using methods that explicitly consider both mean-field and local contributions to polarization. In particular, continuum dielectric models are inadequate for this purpose because they cannot account for local effects and therefore significantly underestimate the degree of polarization of the molecule. These observations have profound consequences for the development, validation, and testing of nonpolarizable models.

## 1 Introduction

Describing electrostatic interactions between molecules is one of the most crucial steps in the development of classical nonpolarizable force fields for condensed phases. Although more rigorous and concomitantly more computationally expensive approaches have been explored (see, e.g., refs (1, 2) and references therein), assigning partial point charges to individual interaction sites followed by the application of Coulomb’s Law has remained the most commonly used method for describing electrostatics, particularly in generic force fields that cover a wide variety of compounds (e.g., CGenFF,^3^ GAFF,^4^ OPLS-AA,^5^ TraPPE^6^). A panoply of methods to assign point charges from quantum mechanical (QM) calculations have been proposed over the years (see, e.g., refs (7−9) and references therein), yet their relative merits are still the subject of intense debate, mostly due to the fact that point charges are not experimental observables, and therefore it is hard to benchmark them independently of other force field parameters.^10^ Furthermore, induction or polarization interactions have to be included implicitly in the parameterization of nonpolarizable models for condensed phases, which significantly complicates the matter. In this context, an alternative approach would be to explicitly describe polarization interactions through a fully polarizable model. Although polarizable force fields are, in principle, more rigorous and have been recently shown to yield improved predictions of the structural, thermodynamic, and dynamic properties of water,^11,12^ this comes at a significantly higher computational cost. The aim of this paper is not to propose an alternative to polarizable models but to discuss how to get the best out of the computationally cheaper approach based on nonpolarizable fixed-charge models, which are the most widely used models and are still at the forefront of force field development.^13^

In the initial efforts to develop nonpolarizable force fields for liquids and solutions during the 1990s, point charges were mostly assigned on the basis of gas-phase QM calculations on prototypical molecules of interest. In recognition of the fact that the model should be somewhat more polarized in the liquid phase than in the gas phase, force field developers chose to use the Hartree–Fock (HF) method^14,15^ with the 6-31G* basis set,^16^ which was known to over-polarize molecules, and was hence assumed to yield charges that were more appropriate to the liquid phase.^17^ This approach is still widely employed and is at the heart of perhaps the two most popular methods for point charge assignment in generic force fields: RESP^17^ and AM1-BCC.^18^ The limitations of this approach, however, have become increasingly apparent, as in the recent benchmark study of Zhou et al.,^19^ which showed that HF/6-31G* charges generally underestimated the degree of polarization in the liquid state and led to inconsistent levels of polarization across different types of molecules.

The inadequacy of the HF/6-31G* approach has led researchers to develop more rigorous ways of describing the correct polarization level of molecules in the liquid state for nonpolarizable models. One option that has been pursued is to scale the charges from a gas-phase QM calculation by an empirical scaling factor. In this case, in contrast with the HF/6-31G* approach, charge calculation methods are selected and/or adjusted to yield accurate gas-phase dipole moments of the molecule in question. The charges are then scaled upward by a uniform factor in an effort to describe a degree of polarization more appropriate for condensed-phase simulations. Although this approach has had some success in predicting hydration free energies^20,21^ and pure liquid properties,^22^ it was found that the value of the scaling factor (normally around 1.20) depends on the details of the charge determination method as well as on the type of solvent.^22^

A more theoretically rigorous approach can be termed the “halfway-charge” method. When a molecule is transferred from the gas phase to the liquid phase, it is polarized by the surrounding medium, which distorts the wave function of the molecule from its equilibrium state in the gas. This carries an energy penalty, commonly referred to as the distortion energy. However, this is more than compensated by a favorable energy term coming from the interactions between the polarized wave function of the molecule with its surrounding liquid-phase environment—the so-called stabilization energy.^23^ It can be shown^24^ that, within a linear response approximation, the favorable stabilization energy is approximately twice as large, in magnitude, as the unfavorable distortion energy, leading to a total polarization or induction energy that is favorable and equal to half of the stabilization energy. When developing a multipole model for water, Karamertzanis et al.^1^ made use of this theory to propose the idea of using point charges that yielded dipole (or multipole) moments that were halfway between those of the gas and the real liquid phase, therefore implicitly capturing the total polarization energy contribution.

This approach was later generalized by Cerutti et al.^25^ to develop a point charge determination scheme, which they named IPolQ, for a newly parameterized version of the Amber FF. In IPolQ, two charge determination procedures are carried out at a high level of QM theory (MP2/cc-pV(T+d)Z)—one for the isolated solute molecule under vacuum and another for the solute molecule surrounded by a liquid-phase electrostatic environment—and the final “halfway-polarized” charges are calculated as the average between the gas- and liquid-phase charges. Since they were interested in calculating hydration free energies, Cerutti et al. chose water for the surrounding liquid environment in the second step of their procedure. Importantly, they described the electrostatic field acting on the solute molecule by a set of explicit point charges obtained from MD simulations, similar to what is done in QM/MM calculations.^26^ The surrounding charges were adjusted to correspond to a “polarized version” of the TIP4P-Ew water model^27^—i.e., they increased the value of the charges so that the dipole moment of the surrounding water molecules was greater than that of normal TIP4P-Ew by the same degree that TIP4P-Ew exceeds the dipole moment of water vapor.^25^ As we have shown in recent calculations of liquid-phase dipole moments using the Self-Consistent Electrostatic Embedding (SCEE) method,^28,29^ this explicit treatment is necessary to provide an accurate representation of the polarization environment induced by polar solvents. Indeed, the water dipole moment used by Cerutti et al. for their polarized version of TIP4P-Ew was 2.78 D,^25^ in excellent agreement with the value of 2.76 ± 0.02 D obtained for liquid water using the most recent version of SCEE.^29^

Muddana et al.^30^ proposed a simplified version of IPolQ, which they called IPolQ-Mod, with the aim of reducing the complexity and computational expense of the calculation of liquid-phase charges. Instead of relying on explicit point charges with positions sampled from MD simulations to describe the electrostatic field of the surrounding solvent, IPolQ-Mod made use of an implicit solvent model, namely, the polarizable continuum model (PCM),^31^ with a dielectric constant equal to that of liquid water. Although the two methods are similar, the distinction is an important one—IPolQ-Mod assumes that a dielectric continuum model is able to provide an accurate representation of the real polarization environment of the liquid state. This has been shown to be a rather crude approximation since continuum models neglect local interactions such as hydrogen bonds and hence lead to a significant underestimation of the degree of polarization in polar solvents.^28,29,32−34^ Furthermore, hydration free energy predictions obtained with IPolQ-Mod charges were shown to be inconsistent with those of the more rigorous reference-potential method at the same level of theory^35^ since the latter also uses an explicit representation of the surrounding solvent environment. Perhaps (at least partly) for this reason, IPolQ-Mod charges do not seem to lead to significant improvements over “standard” HF/6-31G*-based charges in predicting the hydration free energy of small organic solutes.^30,36,37^

Cole et al.^38^ adopted a similar approach when developing a new method for classical force field parameterization based on atoms-in-molecule (AIM) electron density partitioning, which they termed the QUantum mechanical BEspoke (QUBE) force field.^39^ Instead of using transferable atom types to describe a wide range of molecules, individual molecules are instead parameterized directly from QM calculations, with both charges and repulsion/dispersion parameters determined from AIM analysis with only a very small number of adjustable parameters. In their approach, point charges were assigned based on QM calculations of the target molecule in a dielectric continuum model,^40^ but with a dielectric constant ε = 4, chosen such that the favorable and unfavorable contributions to the polarization energy canceled out (we discuss this issue in more detail in Section 3.1). The authors showed that this choice of dielectric constant led to dipole moments on the solutes that were, on average, approximately halfway between those of the gas and the liquid. However, it is important to note that their reference state for the liquid-phase dipole moment was, once again, obtained from a dielectric continuum model with ε = 80 to represent water. As we discussed above, this is known to significantly underestimate the extent of polarization, particularly for strongly polar and hydrogen-bonding fluids.

The idea that point charges should be intermediate between those of the gas and liquid phases is also at the heart of the recently proposed RESP2 method of force field charge assignment.^10^ In this method, point charges are computed as linear combinations of gas- and liquid-phase charges, but the scaling factor is adjusted by fitting against experimental liquid-state properties. As in the IPolQ-Mod and QUBE approaches, the reference liquid state was again an implicit solvent model representing water (i.e., with ε = 78.4). Interestingly, the authors found that the optimal scaling factor for the model point charges was between 0.5 and 0.7, i.e., somewhat larger than the exact “halfway” point employed in all of the previous approaches. This result may, however, reflect the underestimation of the degree of polarization of the liquid state caused by the use of a continuum dielectric model.

A conceptually different approach to the “halfway-charge” idea is based on the application of *post facto* polarization corrections to phase-change energies (e.g., enthalpy of vaporization, solvation free energy). The first authors to apply this type of correction in force field development were Berendsen et al.^41^ when proposing the widely used SPC/E water model. They developed a simple expression to account for the distortion energy of polarization based on the difference between the gas and liquid dipole moments, which has since been generalized by Swope and co-workers.^42^ However, they assumed that the dipole moment of the classical nonpolarizable model was identical to that of the real liquid, an assumption that we now know to be invalid.^28,29^ Indeed, this leads to a significant underestimation of the distortion contribution to the polarization energy.^43−45^ Moreover, the distortion energy is offset by a favorable contribution that is also not accounted for in nonpolarizable force fields—the interaction energy between the fully polarized molecule and the electronic clouds of the surrounding liquid molecules. Effectively accounting for this purely electronic contribution to polarization is the main aim of the MDEC (Molecular Dynamics in Electronic Continuum) framework of Leontyev and Stuchebrukhov.^44^ It turns out that these two contributions (positive distortion and negative electronic polarization) tend to cancel out almost exactly in polar fluids, leading to net polarization energy corrections that are quite small or even negligible.^43−45^

The MDEC theory predicts that the effective dipole moment of a nonpolarizable model is scaled down from the real liquid dipole moment due to dielectric screening by the electronic degrees of freedom of the solvent. The scaling factor is predicted to be  for spherical ions, where ε_∞_ is the high-frequency dielectric constant of the solvent, which describes the purely electronic contribution to the dielectric response of the medium, but the exact form of the scaling for non-spherical neutral molecules is uncertain. Nevertheless, Leontyev and Stuchebrukhov^45^ demonstrated that the MDEC theory leads to effective dipole moments of water that are consistent with empirically fitted values used in most fixed-charge water models, which suggests that the charge scaling may emerge spontaneously when fitting a force field to experimental data. An important consequence of scaling the charge to best describe the potential energy surface (PES) of a liquid, thereby implicitly accounting for the effects of polarization, is that the nonpolarizable model is not able to simultaneously describe the PES and the dipole moment surface (DMS) since describing the latter would require the molecule to be fully polarized as in the real liquid. This means, as cogently argued by Vega,^46^ that properties that depend directly on the DMS, such as the dielectric constant, cannot be accurately predicted by effective fixed-charge models fitted to describe the PES. One can, however, derive a *post facto* correction for the dielectric constant that has been shown to eliminate systematic deviations observed in predictions of popular nonpolarizable force fields.^47,48^ Recently, these ideas—charge scaling of the model dipole moment and *post facto* corrections for phase-change energies and dielectric constant—have been incorporated for the first time into the force field parameterization workflow, leading to the Polarization-Consistent Approach (PolCA) force field for alcohols.^49^

In summary, although they are grounded on somewhat different theoretical principles, both the “halfway-charge” approach and the MDEC theory make two key predictions that are consistent: (1) to correctly describe the PES of the liquid phase, the effective point charges of nonpolarizable models need to be intermediate between those of the gas and the liquid state and (2) this charge scaling leads to net polarization corrections to the energy that are very small or zero. However, demonstrating the validity of these two predictions and their impact on the accuracy of force field predictions has been hampered, on the one hand, by the coupling between electrostatic and non-electrostatic parameters of the force field and, on the other hand, by the difficulties in obtaining a reliable estimate of the dipole moment (and hence the degree of polarization) of real liquids.

In this paper, we attempt to reconcile the above two theoretical frameworks and demonstrate that the dipole moment of classical nonpolarizable force fields should indeed be halfway between the gas and liquid dipole moments. We make use of accurate values for the liquid-phase dipole moments of water and methanol determined using our recently developed SCEE method^29^ as reference points to develop automated force fields for both compounds based on the QUBE approach of Cole et al.^38,39^ We show that when the correct liquid-phase dipole moments are used, the predicted enthalpy of vaporization is independent of the choice of model dipole moment, provided the corresponding polarization corrections are applied, thus validating the theory of Karamertzanis et al.^1^ and Cerutti et al.^25^ However, if the liquid-phase dipole moment is estimated from a continuum dielectric model (as done in the IPolQ-Mod, QUBE, and RESP2 approaches), which does not fully capture the extent of liquid polarization, the enthalpy of vaporization is *not* independent of the choice of model charges. Furthermore, we show that in the former case, the enthalpy of vaporization can be predicted directly from MD simulations without the application of *post facto* polarization corrections when the model dipole moment is precisely the average of the gas and liquid moments. We provide further proof for this hypothesis by carrying out force field optimizations following the PolCA protocol,^49^ where the model dipole moment is varied uniformly over a wide range, and the remaining nonbonded parameters are optimized to match experimental data. We observe that the residual of the fit against experimental data (which includes the enthalpy of vaporization) goes through a minimum very close to the halfway point between the gas and liquid dipole moments. This offers independent validation for the hypothesis that the optimal point charges for use in nonpolarizable force fields of liquids should yield a model dipole moment that lies between the gas and liquid dipole moments, with the latter determined through an appropriate QM method that can account for both mean-field and local contributions to polarization.

## 2 Methodology

With the aim of obtaining independent support for the use of half-polarized charges in liquid-phase nonpolarizable models, we make use of two separate force field parameterization approaches: (i) the QUantum mechanical BEspoke (QUBE) force field^38^ and associated toolkit (QUBEKit)^39^ developed by the Cole group and (ii) the Polarization-Consistent Approach (PolCA) force field developed by the Jorge group.^49^ The QUBE force field is based on the concept of QM-to-MM parameter mapping, which essentially tries to reduce the number of empirical fitting parameters to a minimum by parameterizing as many components as possible directly from QM calculations on individual molecules. PolCA, in contrast, has a strong component of empirical parameter fitting, making use of advanced optimization techniques to try to accurately describe as many thermodynamic properties of the target liquids as possible. Crucially, however, it has (for the first time, to our knowledge) incorporated the use of *post facto* polarization corrections and charge scaling into the force field parameterization workflow. The two force field approaches, as well as the corresponding technical details, are described in separate subsections.

### 2.1 QUBE Force Field

Force field parameters for water and methanol were derived using the QUBEKit software package, using methods described in detail elsewhere.^39^ In brief, the ground state electron densities were computed using the ONETEP density functional theory code,^50^ with the Perdew–Burke–Ernzerhof (PBE) exchange–correlation functional. The DDEC module in ONETEP^51^ was used to partition the total electron density and assign atomic charges and volumes. Lennard–Jones (σ and ε) parameters were estimated from the atomic volumes using rescaling protocols based on the Tkatchenko–Scheffler method.^52^ Both water and methanol were described using an all-atom (AA) approach. However, the dispersion/repulsion contributions of the polar hydrogen atoms were included in the adjacent oxygen atom, and the Lennard–Jones parameters of hydroxyl and water hydrogens were set to zero.^38^ Furthermore, point charges were placed at the center of each interacting atom; even though off-center point charges have previously been used in models for both these molecules and are known to lead to generally better predictive performance, this was not attempted here for simplicity. We do not expect our conclusions to depend on the use of virtual charged sites. QUBE models were generated for both water and methanol using different values of the dielectric constant of the continuum solvent model,^40^ ranging from 1 (corresponding to vacuum) to 80 (representing liquid water). Tables 1 and 2 report the nonbonded parameters generated for each dielectric constant. Bonded parameters (which are independent of the solvent dielectric constant) were computed using the modified Seminario method^53^ and are reported in Tables S1 and S2. However, it should be noted that we have adopted a fully rigid description of the water molecule for simplicity and to allow a more direct comparison with widely used fixed-charge rigid water models. The flexibility of the model has a negligible effect on the enthalpy of vaporization, which is the main property of concern to us (see below).

**Table 1:** QUBE Nonbonded Parameters for Methanol, Derived from QM Calculations Using Different Values of the Surrounding Continuum Dielectric Constant (ε)^a^

ε	μ_QM_	*q*_O_	*q*_Ho_	*q*_C_	*q*_Hc_	σ_O_	ε_O_	σ_C_	ε_C_	σ_Hc_	ε_Hc_
1	1.581	–0.5972	0.3875	0.1117	0.0327	0.3085	0.5454	0.3236	0.2592	0.2479	0.1505
2	1.696	–0.6196	0.4019	0.1150	0.0342	0.3103	0.5344	0.3231	0.2592	0.2475	0.1505
3	1.762	–0.6335	0.4108	0.1171	0.0352	0.3114	0.5278	0.3229	0.2592	0.2473	0.1505
4	1.805	–0.6424	0.4166	0.1184	0.0358	0.3121	0.5239	0.3227	0.2592	0.2472	0.1505
5	1.836	–0.6488	0.4208	0.1194	0.0362	0.3126	0.5210	0.3226	0.2592	0.2471	0.1505
10	1.921	–0.6665	0.4325	0.1219	0.0374	0.3139	0.5133	0.3223	0.2592	0.2469	0.1505
20	1.991	–0.6816	0.4425	0.1241	0.0383	0.3150	0.5076	0.3221	0.2592	0.2469	0.1505
40	2.052	–0.6954	0.4519	0.1260	0.0392	0.3159	0.5029	0.3220	0.2592	0.2469	0.1505
80	2.109	–0.7087	0.4610	0.1280	0.0399	0.3167	0.4990	0.3218	0.2592	0.2471	0.1505

a Subscripts for the point charges (*q*_i_) and Lennard–Jones parameters (σ_i_ and ε_i_) are: O for oxygen, C for carbon, Ho for the hydroxyl hydrogen, and Hc for the aliphatic hydrogens. Also shown are the dipole moments of methanol obtained from the corresponding QM calculation (μ_QM_). Charges are in a.u., σ is in nm, and ε is in kJ/mol.

**Table 2:** QUBE Nonbonded Parameters for Water, Derived from QM Calculations Using Different Values of the Surrounding Continuum Dielectric Constant (ε)^a^

ε	μ_QM_	*q*_Ow_	*q*_Hw_	σ_Ow_	ε_Ow_
1	1.808	–0.8321	0.4160	0.3180	0.9649
2	1.927	–0.8630	0.4315	0.3201	0.9038
3	1.991	–0.8822	0.4411	0.3214	0.8642
4	2.032	–0.8946	0.4473	0.3222	0.8411
5	2.062	–0.9035	0.4518	0.3228	0.8254
10	2.143	–0.9281	0.4640	0.3244	0.7840
20	2.213	–0.9490	0.4745	0.3256	0.7546
40	2.275	–0.9681	0.4841	0.3268	0.7301
80	2.336	–0.9869	0.4934	0.3279	0.7097

a Subscripts for the point charges (*q*_i_) and Lennard–Jones parameters (σ_i_ and ε_i_) are: Ow for oxygen, Hw for hydrogen. Also shown are the dipole moments of water obtained from the corresponding QM calculation (μ_QM_). Charges are in a.u., σ is in nm, and ε is in kJ/mol.

For each model reported in Tables 1 and 2, we carried out MD simulations of methanol and water, respectively, both in the gas phase and in the liquid phase. The liquid simulations were carried out in cubic simulation boxes with periodic boundary conditions applied in all three dimensions. We used 500 molecules for methanol, yielding simulation boxes of lengths between 3.2 and 3.3 nm, while the water simulations contained 900 molecules, yielding boxes of lengths ∼3.1 nm. We used a time step of 2 fs with the Verlet leapfrog integrator.^54^ The temperature was kept constant at 298 K using a Nosé–Hoover thermostat^55,56^ while the pressure was fixed at 1 bar using the Parrinello–Rahman barostat.^57^ A cut-off of 1.2 nm was used for the Lennard–Jones potential, with long-range dispersion corrections added to both energy and pressure, while long-range electrostatic interactions were accounted for using the particle-mesh Ewald (PME) method.^58^ Gas-phase MD simulations made use of the same protocol, except that the simulation boxes contained a single molecule and no periodic boundary conditions or cut-off radius were applied, hence replicating a vacuum environment. Furthermore, no barostat was applied in the gas-phase simulations. Liquid simulations were run for 10 ns, while vacuum simulations were run for 20 ns. All MD simulations were carried out with GROMACS software, version 5.1.2.^59,60^ The enthalpy of vaporization was computed for each model from the potential energy in the gas and liquid simulations, according to
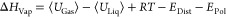
1In eq 1, *U*_Liq_ is the molar potential energy in the liquid phase, *U*_Gas_ is the potential energy in the gas phase, and the angular brackets denote ensemble averages. *E*_Dist_ is a correction term accounting for the electronic distortion of the molecule when moving from the gas to the liquid phase (hence it is a positive value), and *E*_Pol_ is a correction term to account for the favorable interactions between the polarized molecule and the surrounding liquid (hence it is a negative value). The physical meaning of the two correction terms will be described below in further detail.

### 2.2 PolCA Force Field

The PolCA force fields for methanol and all other alcohols employed a United-Atom (UA) approximation, whereby the contribution of each hydrogen atom was implicitly included in the parameters of the adjacent heavy atom (e.g., the CH_X_ group consists of a single interaction site). In all of the results reported herein, only the Lennard–Jones (LJ) parameters of the hydroxyl oxygen atom were optimized. Parameters for the alkane CH_X_ groups were taken from a previous model designed to accurately reproduce bulk liquid properties and self-solvation free energies of alkanes.^61^ As in our previous PolCA model,^49^ bonded parameters were taken from the TraPPE force field for alcohols,^62^ and all bonds were kept rigid. The point charges for the hydroxyl group and the adjacent CH_X_ site (charges on remaining alkyl sites are set to zero by construction) were kept fixed during each parameter optimization, but different charge sets were used, as described below.

Force field parameters were optimized against experimental data for the density, enthalpy of vaporization, and self-diffusion coefficient of methanol, using a similar optimization approach as described in the original PolCA publication,^49^ and the reader is referred there for further details. Metamodels were used to predict molecular simulation results for a given set of parameters since they have been found to reduce computational expenses and allow for more efficient exploration of the parameter space.^63^ The precise way in which each metamodel was constructed from a grid of MD simulation results is described in the Supporting Information (SI, Section S2). The objective function was defined using eq 2, where *k* is the target property, *f*_k_(*x*_1_,*x*_2_) is the value predicted by the metamodel, and *y*_exp_*k*__ is the experimental value.
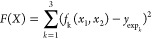
2

This function was minimized using the steepest descent algorithm with a variable step length and a maximum number of iterations equal to 4000. The lowest value from these iterations was used as the initial point for a second optimization which used smaller step lengths and a maximum number of iterations equal to 100.

We started by developing three models for methanol, based on three different charge sets: (1) charges obtained from a QM calculation carried out under vacuum (i.e., “gas-phase” charges); (2) charges obtained from a QM calculation carried out in a dielectric continuum and scaled so that the methanol dipole moment was the same as the SCEE liquid-phase value^29^ (i.e., “liquid-phase” charges); and (3) charges obtained from a QM calculation carried out in a dielectric continuum and scaled so that the methanol dipole moment was exactly the average between the gas-phase and liquid-phase SCEE dipole moments (i.e., “halfway-polarized” charges). As explained previously, the LJ parameters of the hydroxyl oxygen atom were optimized to yield the best match against experimental density, enthalpy of vaporization, and self-diffusion coefficient of methanol. This optimization step was carried out separately for each of the three charge sets. MD simulations were then performed for the entire series of linear alcohols, from methanol to 1-decanol, from which pure liquid properties were calculated.

The MD simulation protocol was identical to that used in our previous work.^49^ In brief, simulations were carried out with GROMACS version 5.1.2^59,60^ using a time step of 2 fs and the Verlet leapfrog integrator.^54^ Each bulk liquid simulation was run up to 25 ns, making use of fully periodic cubic boxes, a V-rescale thermostat^64^ to keep the temperature at 298 K, a Parrinello–Rahman barostat^56^ to fix the pressure at 1 bar, a cut-off of 1.0 nm for the LJ interactions, together with long-range dispersion corrections, and the PME method^58^ to handle long-range electrostatics. Liquid simulation boxes were packed with the required number of molecules for each alcohol so as to keep the box length at around 3 nm after equilibration (see ref (49) for the exact number of molecules used in each simulation). Gas-phase simulations were run up to 50 ns with a V-rescale thermostat^64^ to keep the temperature at 298 K but without periodic boundary conditions or a cut-off radius. The liquid density was calculated from the average volume at equilibrium using the *gmx energy* tool. The enthalpy of vaporization was calculated using eq 1, except that no polarization corrections were applied with the aim of testing the validity of the “halfway-charge” approach. The self-diffusion coefficient was calculated from the mean-square displacement using Einstein’s formula, making use of the *gmx msd* tool, with finite-size corrections applied to yield results that are equivalent to macroscopic experiments (see ref (49) for details). The dielectric constant was calculated from the fluctuations in the dipole moment of the simulation box using the *gmx dipoles* tool. A *post facto* correction was applied to the dielectric constant predicted by MD to account for the effects of polarization, as described in detail elsewhere.^47^ Ten replicas of each simulation (liquid and vacuum) were run to obtain results with high precision (this is particularly important for the dielectric constant, as described in ref (49)). Error bars in the plots (when sufficiently large to be visible) report the 95% confidence interval of the mean.

For each of the three models described above, we also carried out simulations of the self-solvation free energy of methanol. We used Bennett’s acceptance ratio (BAR) method^65,66^ to sample the free energies via a one-step transformation using the option “couple-intramol = no” in GROMACS. The LJ component of the free energy was calculated using 15 λ values (0, 0.15, 0.2, 0.25, 0.3, 0.35, 0.4, 0.45, 0.5, 0.55, 0.6, 0.7, 0.8, 0.9, and 1), while seven λ values were used to obtain the electrostatic component (0, 0.3, 0.6, 0.7, 0.8, 0.9, and 1). Each MD simulation was run using the same protocol described above for bulk liquid simulations, except that a stochastic dynamics integrator^67^ and the corresponding Langevin thermostat were employed, and the run length was 5 ns for both the LJ and electrostatic components. During the decoupling of the LJ component, a soft-core function^68^ was applied to avoid instabilities close to the noninteracting state, with parameters sc-power = 1, sc-sigma = 0.3, and sc-α = 0.5. The above protocol was found to yield accurate results for solvation free energies with a relatively modest computational expense.^49^

Finally, we also carried out a series of optimizations starting from several different initial charge sets—see Table 3 for the initial charge values—determined using a variety of approaches ranging from “pure” QM calculations to those taken from empirically fitted models. Specifically, we used methanol charges from two (mainly) empirical force fields, OPLS-AA^69^ and TraPPE,^62^ as well as from two literature charge calculation approaches—the AM1-BCC method^70,71^ is used in a wide variety of standard force fields, including GAFF, while the recent IPolQ method^25^ employs the halfway-charge approach with a fully polarized liquid reference state (see Section 1). We also carried out our own QM calculations for methanol in an IEFPCM dielectric continuum model^72^ with default parameters as implemented in Gaussian09.^73^ From that same underlying QM calculation, we extracted two sets of point charges, one using CHelpG^74^ and another using the more recent DDEC method,^75^ thought to provide a good balance between an accurate description of the electrostatic potential and chemically realistic point charges. Note that we also carried out QM calculations for methanol using the SMD solvation model,^76^ but those differed from PCM charges by a constant scaling factor; therefore, they were not included in the analysis. For each of the initial charge sets shown in Table 3, we scaled the point charges uniformly using a scaling factor α to yield models with a wide range of dipole moments, ranging from the gas-phase to the liquid-phase values for methanol (i.e., ∼1.65 to ∼2.6 D). For each value of α, we optimized the LJ parameters of the hydroxyl oxygen atom using the optimization procedure described above and in the SI, thus obtaining “scans” of the model dipole moment variable for each model. We then compared the values of the minimized objective function to find the optimal model dipole moment corresponding to each charge set, as discussed in detail in Section 3.2.

**Table 3:** Initial Point Charge Sets and Corresponding Dipole Moments Used for the “Scanning” Optimizations, whereby the Model Dipole Moments were Varied over a Wide Range from the Gas-Phase to the Liquid-Phase Values^a^

charge set	μ_M_	*q*_O_	*q*_Ho_	*q*_CH3_
DDEC	2.07	–0.645	0.415	0.230
CHelpG	2.25	–0.693	0.416	0.277
AM1-BCC	1.93	–0.600	0.397	0.203
IPolQ	2.01	–0.626	0.410	0.216
OPLS	2.21	–0.683	0.418	0.265
TraPPe	2.26	–0.700	0.435	0.265

a Each of the initial charge sets was uniformly scaled by a factor α, while the LJ parameters of the hydroxyl oxygen were optimized.

## 3 Results and Discussion

### 3.1 Self-Consistency of the QUBE Approximation

**Figure 1:**
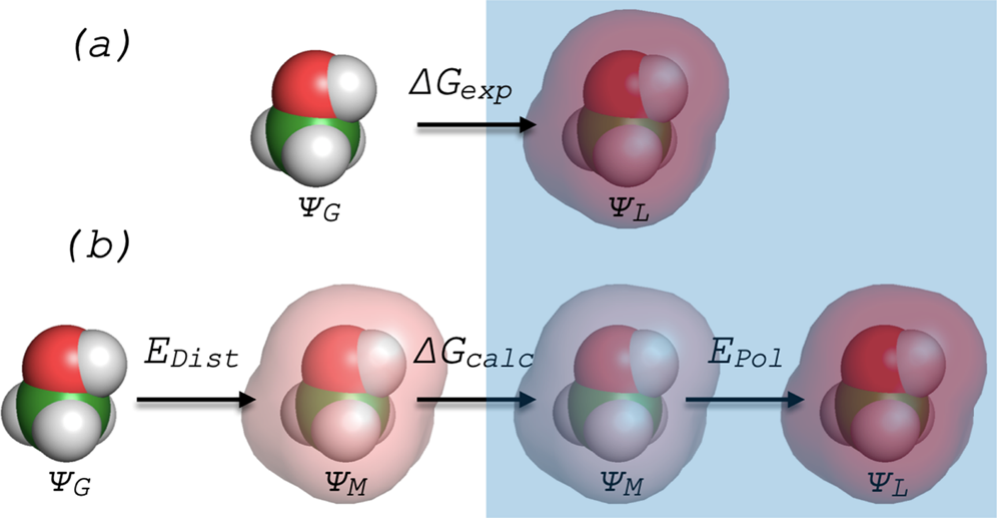
Diagram representing the transfer of a methanol molecule from the gas phase to the pure liquid phase (a). Polarization of the electronic ground state density is denoted by red surfaces. The same process is shown in (b), but now the molecule is transferred through two hypothetical intermediate states in which the ground state electron density is first “half-polarized” under vacuum and then transferred to the liquid phase.

We started by developing QM-based force fields, based on the QUBE approach of Cole et al.,^38^ for both water and methanol. Several models were developed, corresponding to different values of the dielectric constant used in the dielectric continuum calculations. For each of these models, the two polarization energy contributions were calculated as defined in Cole et al.^38^ (see Figure 1). The process of transferring a molecule from the gas state (where its wave function is denoted by Ψ_G_) to a polarized liquid state (with a fully polarized wave function Ψ_L_), shown in Figure 1a, is decomposed into a series of steps via an intermediate “model” state (wave function denoted by Ψ_M_). *E*_Dist_ (the first step in Figure 1b) is the unfavorable distortion energy of polarizing the gas-phase wave function to the intermediate model state, which is carried out under vacuum. The molecule, described by fixed charges corresponding to the intermediately polarized wave function Ψ_M_, is then immersed in the surrounding liquid (second step in Figure 1b), which corresponds to the calculation done in a standard MD simulation with a fixed-charge model. In the third step, *E*_Pol_ is an overall favorable energy, but it comprises two contributions: (i) the distortion energy due to polarizing the molecular wave function from the intermediate model state to the fully polarized liquid state and (ii) the favorable stabilization energy caused by the enhanced interaction between the polarized wave function and the surrounding liquid. This is because the third step in Figure 1b is carried out in the liquid state, so both the above contributions play a role. In these calculations, as discussed previously, the reference state for the liquid was chosen to be a dielectric continuum with a value of 80, approximately matching the experimental value for pure water. The central idea behind the QUBE approach to charge assignment is to find the intermediate level of polarization where *E*_Dist_ + *E*_Pol_ = 0. It can be shown (see the Supporting Information) that, within the linear response approximation, this corresponds to the concept of “halfway-polarized” charges described above.

**Figure 2:**
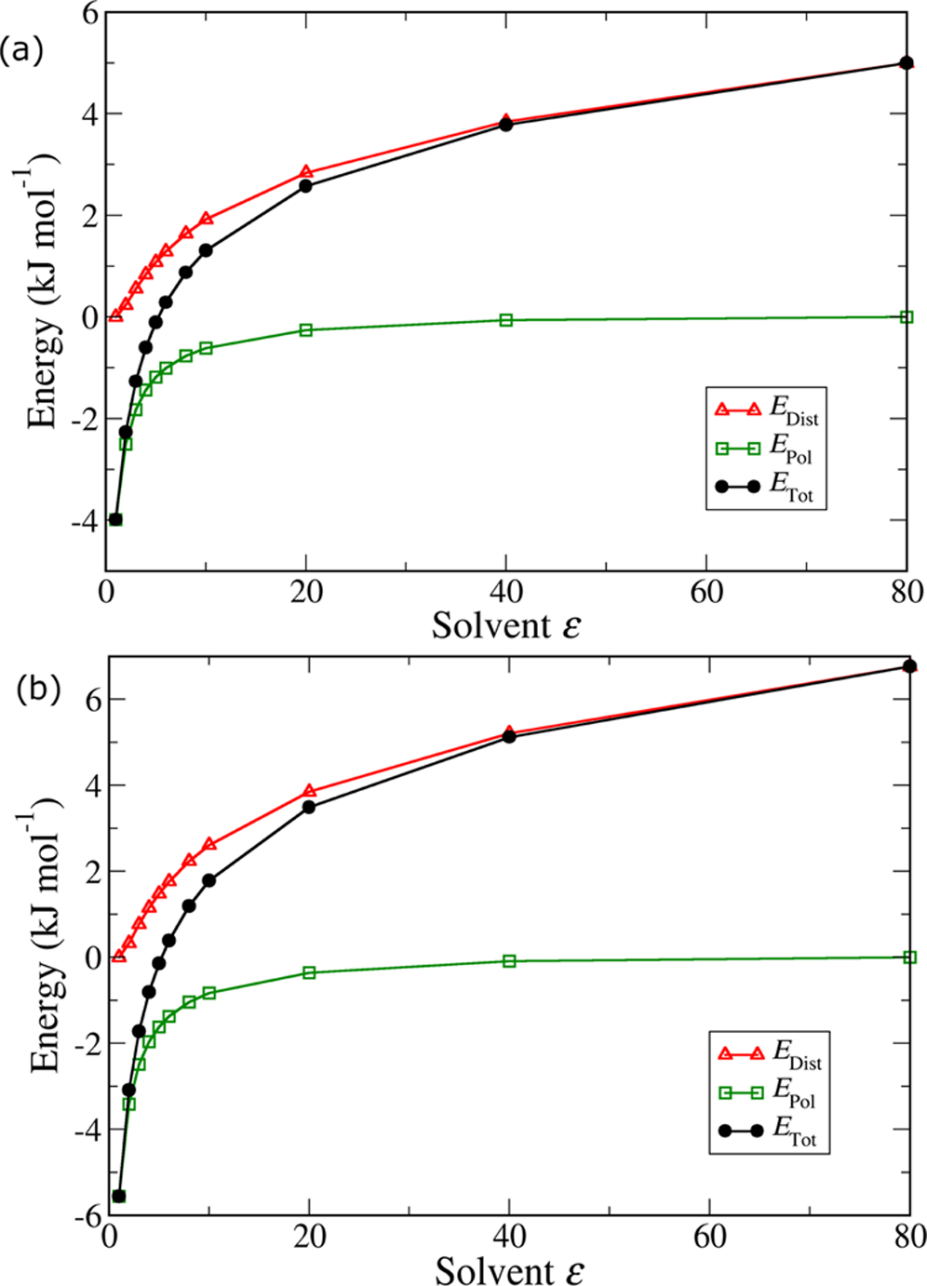
Polarization energy contributions, according to the QUBE approximation, for methanol (a) and water (b) determined from QM calculations on a dielectric continuum as a function of the dielectric constant of the continuum model.

In Figure 2, we show the values of these two energy contributions, as well as their sum, for both water and methanol as a function of the dielectric constant of the surrounding medium. By construction, *E*_Dist_ is zero when the QM calculation is done in the gas phase—i.e., the intermediate state is the same as the gas state, and the model is not prepolarized at all; hence, there is no distortion energy. In contrast, *E*_Pol_ is quite substantial when the model dipole is the same as the gas-phase dipole but tends to zero when the dielectric constant approaches that of the liquid—in that limit, the intermediate state is the same as the liquid state; hence, there is no “post-polarization” step. Between these two limits, both energy contributions are non-zero, as expected. At a certain intermediate value, the two contributions cancel out, and the net polarization energy is zero. For the particular cases of water and methanol, this happens when ε ∼ 5—we recall that a compromise value of ε = 4 was adopted by Cole et al.^38^ based on their simultaneous analysis of several compounds. We also note that the QM calculations with ε = 5 yield dipole moment values that are very close to the average between the gas and liquid dipoles for both compounds (see Tables 1 and 2), as expected from linear response theory.

As with all methods based on the “halfway-charge” concept, the model point charges are chosen in QUBE so that the net polarization energy is zero, and hence no corrections need to be added to the results of MD simulations. However, an implicit assumption in the scheme described in Figure 1 is that the same results should be obtained using *any* set of charges between the gas and liquid levels of polarization, provided the appropriate corrections are added to the MD results. For example, one might choose an over-polarized charge set obtained with, say ε = 20, run an MD calculation of the enthalpy of vaporization, and then subtract^77^ the corresponding polarization energy described by *E*_Dist_ + *E*_Pol_ for that value of ε (which in this particular case would be a positive value; see Figure 2). For the procedure to be self-consistent, the results for the enthalpy of vaporization should be independent of the dielectric constant used to determine the point charges. In what follows, we aim to verify the validity of this assumption.

**Figure 3:**
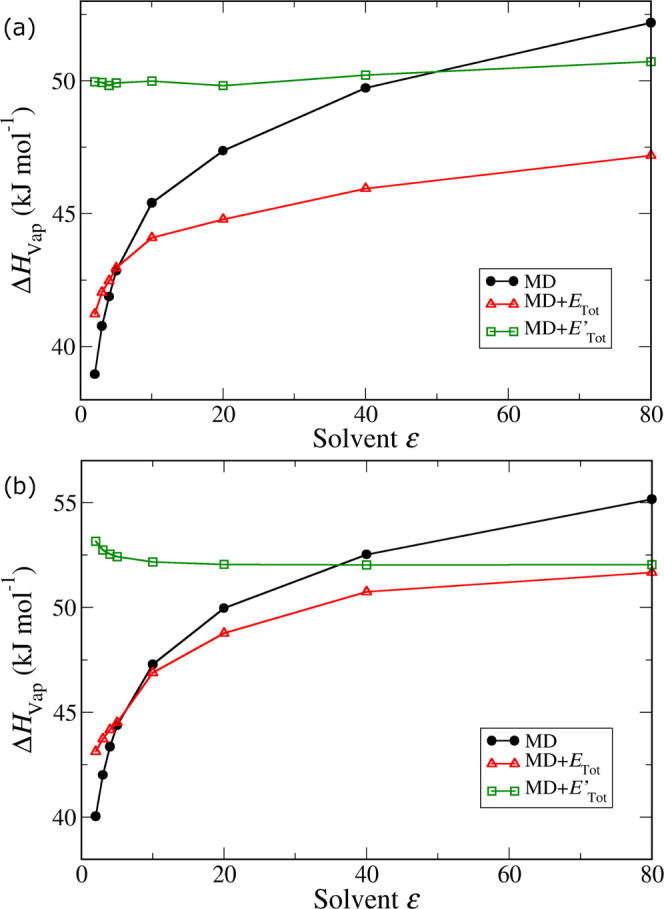
Enthalpies of vaporization for methanol (a) and water (b) determined from MD simulations of several models, with point charges calculated using QM calculations on a dielectric continuum as a function of the dielectric constant of the continuum model. The black curve corresponds to the uncorrected results obtained from MD, the red curve includes the polarization corrections determined using the original approach of Cole et al.^38^ (i.e., when the reference liquid dipole moment was estimated from a QM calculation on a dielectric continuum), and the green curve includes estimated polarization corrections when the reference liquid dipole moment is calculated using the SCEE method.

For each of the models developed above (Tables 1 and 2), we carried out MD simulations of both pure liquids, water and methanol, and computed the enthalpy of vaporization. We then subtracted^77^ the corresponding polarization correction (which was close to zero for ε = 5, as explained above, but non-zero otherwise), thus estimating the “experimental” enthalpy of vaporization corresponding to each model. The results of this analysis are plotted in Figure 3 as the red curve, while the original uncorrected values from MD are plotted as the black curve. While the application of polarization corrections makes the vaporization enthalpies less dependent on the model dipole moment, it is clear that there is still a strong dependence for both molecules. This means that either the underlying assumption regarding the cancelation of the polarization correction is invalid or the liquid-phase dipole moment is inaccurate.

To test the latter hypothesis, we have calculated^29^ the liquid-phase dipole moment of water and methanol using the SCEE method, which is able to account for both mean-field and local (e.g., hydrogen bonding) contributions to the polarization in the liquid phase. The values are 2.759 D for water and 2.606 D for methanol, which are both much higher than the corresponding estimates obtained in the pure dielectric continuum—2.336 D for water and 2.109 D for methanol. As discussed in detail elsewhere,^28,29^ continuum models strongly underestimate the liquid dipole moment since they are not able to account for local contributions in an accurate way. This already indicates that a potential explanation for the inconsistency discussed above is due to the strong underestimation of the liquid dipole moment used as a reference state in the calculations.

Ideally, we would like to recalculate the two polarization energies, still following the scheme in Figure 1, but now using the SCEE wave function as a reference for the liquid state. Note that it is only *E*_Pol_ that is affected by this change in the reference state, while *E*_Dist_ remains unaffected—the reference state for the latter is the gas-phase wave function. Unfortunately, because the dielectric continuum model is unable to polarize the QM molecule to the more realistic levels obtained with SCEE, we are unable to directly calculate *E*_Pol_ for higher dipole moment values. Instead, we estimate it using a fitting procedure.

**Figure 4:**
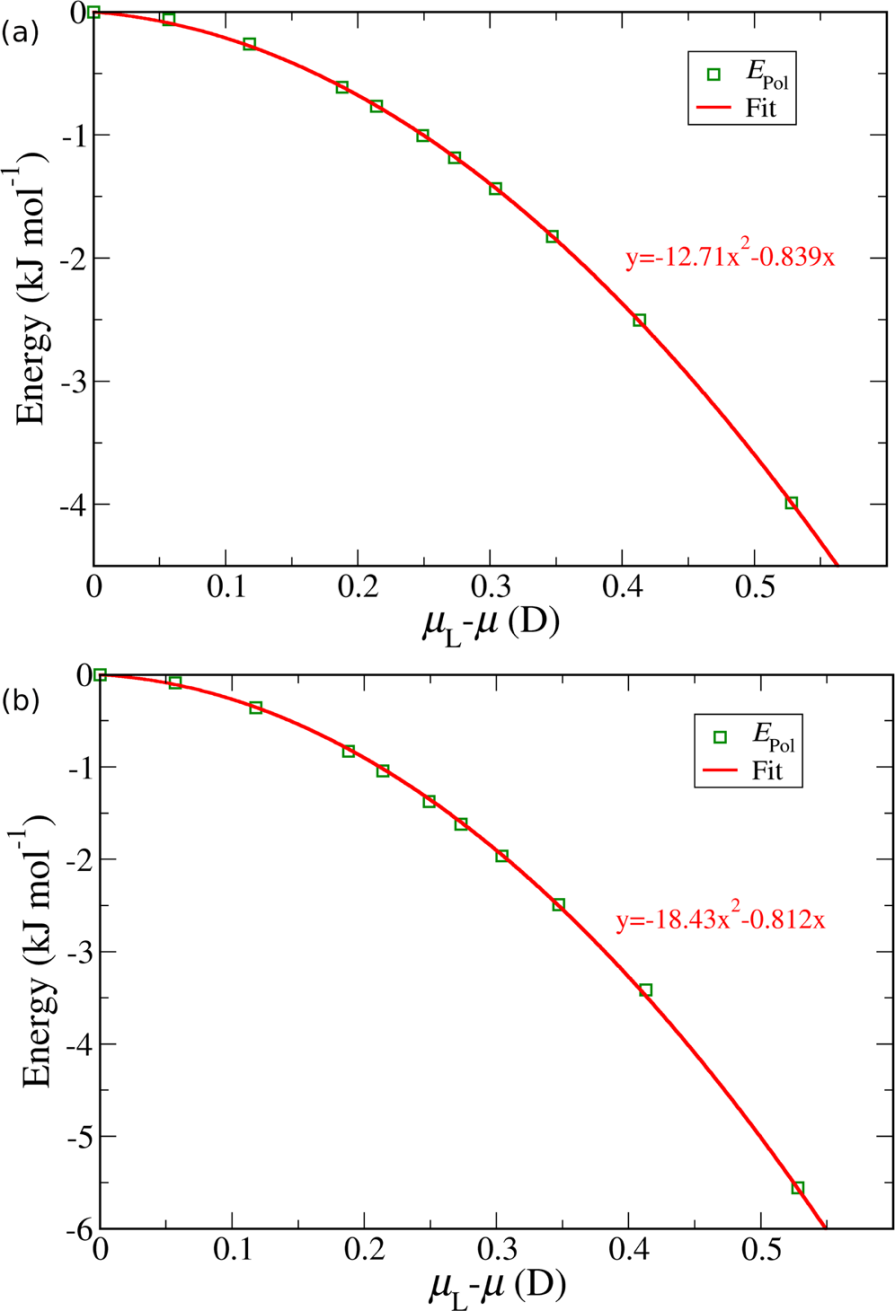
Polarization energy contribution for methanol (a) and water (b) as a function of the difference between the reference liquid-phase dipole moment and the instantaneous QM dipole moment, obtained from a dielectric continuum model. The lines show fits to a second-order polynomial.

We started by replotting the data for *E*_Pol_ (green lines in Figure 2) as a function of the difference between the reference liquid dipole moment (in this case, the dielectric model with ε = 80) and the instantaneous dipole moment obtained from each QM calculation. As shown in Figure 4, *E*_Pol_ is zero when the QM calculation is the same as the reference liquid state and is strongly negative when the QM calculation is done in the gas phase. We then fit a second-degree polynomial through the data (red lines in Figure 4). This quadratic dependence of the polarization energy on the dipole moment shift is expected from prior theoretical treatments of polarization^24,42,44^ (see the Supporting Information for details). As can be seen, the fits are excellent, allowing us to extrapolate the polarization energy contributions to higher levels of polarization. More precisely, we recalculate μ_L_—μ using the SCEE dipole moment for the reference liquid instead and then estimate *E*_Pol_ from the correlation *E*_Pol_ = *f*(μ_L_—μ) given in Figure 4. This leads to much higher magnitudes of *E*_Pol_ because the reference liquid dipole moment is itself much larger. For example, for the QUBE methanol model with ε = 10 and a model dipole moment of 1.92 D, the original values of *E*_Pol_ and *E*_*Tot*_ were −0.6 and 1.3 kJ/mol, respectively (see Figure 2a), while the new values using the SCEE reference dipole moment are −6.5 and −4.6 kJ/mol, respectively.

Using these estimated polarization energies, together with the original distortion energies, we can recalculate the total polarization corrections to the enthalpy of vaporization but now take the more correct SCEE calculation as a reference for the liquid-phase dipole moment. This is shown as the green points in Figure 3. Remarkably, the corrected enthalpy of vaporization now becomes practically independent of the model dipole moment, in agreement with the original assumption of Cole et al.^38^ This confirms that the previously observed inconsistency was not due to a breakdown of the underlying assumption but to an incorrect estimate of the liquid-phase dipole moment. Moreover, when using the more accurate liquid reference state, the model dipole moment that corresponds to a total polarization correction of zero (which can be found at the intersection of the green and black curves in Figure 3) is very close to the average between the gas and liquid (SCEE) dipole moments—2.07 vs 2.09 D for methanol and 2.27 vs 2.28 D for water.

The predicted values of the enthalpy of vaporization for methanol and water using the QUBE approach with the new liquid reference state, i.e., at the point where the total polarization energy is zero, are both much higher than the experimental values (see Table 4). As for the predicted densities, while the result for methanol is not far off the experimental value, the water model significantly underestimates the liquid experimental density. Such discrepancies are somewhat to be expected since the repulsion/dispersion parameters for QUBE were trained^39,78^ over a wide range of compounds using the under-polarized liquid reference state (i.e., from a dielectric continuum calculation). Fully incorporating the more accurate reference state for the liquid-phase dipole moment that we propose here into the QUBE workflow would require an SCEE calculation to be carried out on each molecule of the QUBE training set (25 molecules in total), followed by a full refitting of the force field parameters.^78^ Although SCEE provides a good balance between accuracy and computational speed for the calculation of realistic liquid-phase dipole moments, it is still too time-consuming to apply to such a large number of molecules within a reasonable time frame. Therefore, we are currently trying to develop an even faster and fully automated computational approach that can be applied for this purpose, and we expect to report on these developments in the near future. It is important to emphasize that the aim of the present paper is to analyze the internal consistency of the charge determination procedure, not to optimize and test the QUBE force field.

**Table 4:** Density and Enthalpy of Vaporization of Water and Methanol Predicted by the QUBE Force Field, with and without Optimization of the Lennard–Jones Parameters, Compared against Experimental Data

liquid	property	experimental	QUBE original	QUBE re-optimized
water	ρ (kg/m^3^)	997.0	916.9 ± 0.4	1000.5 ± 0.4
	Δ*H*_Vap_ (kJ/mol)	44.0	52.0 ± 0.5	43.2 ± 0.5
methanol	ρ (kg/m^3^)	786.6	782.4 ± 0.5	787.4 ± 0.5
	Δ*H*_Vap_ (kJ/mol)	37.6	50.2 ± 1.0	37.5 ± 1.0

Nevertheless, to demonstrate that accurate predictions can indeed be obtained using the proposed halfway-charge approach, we have optimized the Lennard–Jones parameters of both the water and methanol models using Force Balance^79^ (see Supporting Information Section S5 for details). With relatively small changes in the LJ parameters (see Table S6), we were able to predict the density and enthalpy of vaporization of both fluids accurately (Table 4). This exercise, however, does not yet demonstrate that halfway-polarized charges lead to the *best* performance when the LJ parameters are empirically fitted. We address this point in the following section.

### 3.2 Optimal Dipole from PolCA Optimization

In the previous section, we showed that the “halfway-charge” approach, as implemented in the QUBE force field, leads to an internally consistent way to implicitly account for the energetic effects of polarization, but only when an accurate liquid-phase reference state, where both mean-field and local contributions to the polarization process are accounted for, is employed. In this section, by applying the PolCA force field parameterization approach, we try to assess if halfway-polarized charges are indeed able to provide more accurate predictions of pure liquid and phase-change properties. Although there have been a few recent attempts to answer this question,^10,30,36,37^ none have made use of a fully polarized liquid state when assigning charges—with the exception of the IPolQ method,^25^ which, to our knowledge, has not yet been tested for condensed-phase properties, all other studies made use of dielectric continuum models to estimate the liquid-phase dipole moments and charges. We restrict our study in this section to methanol since alcohols are so far the only class of molecule for which a PolCA force field has been developed.

**Figure 5:**
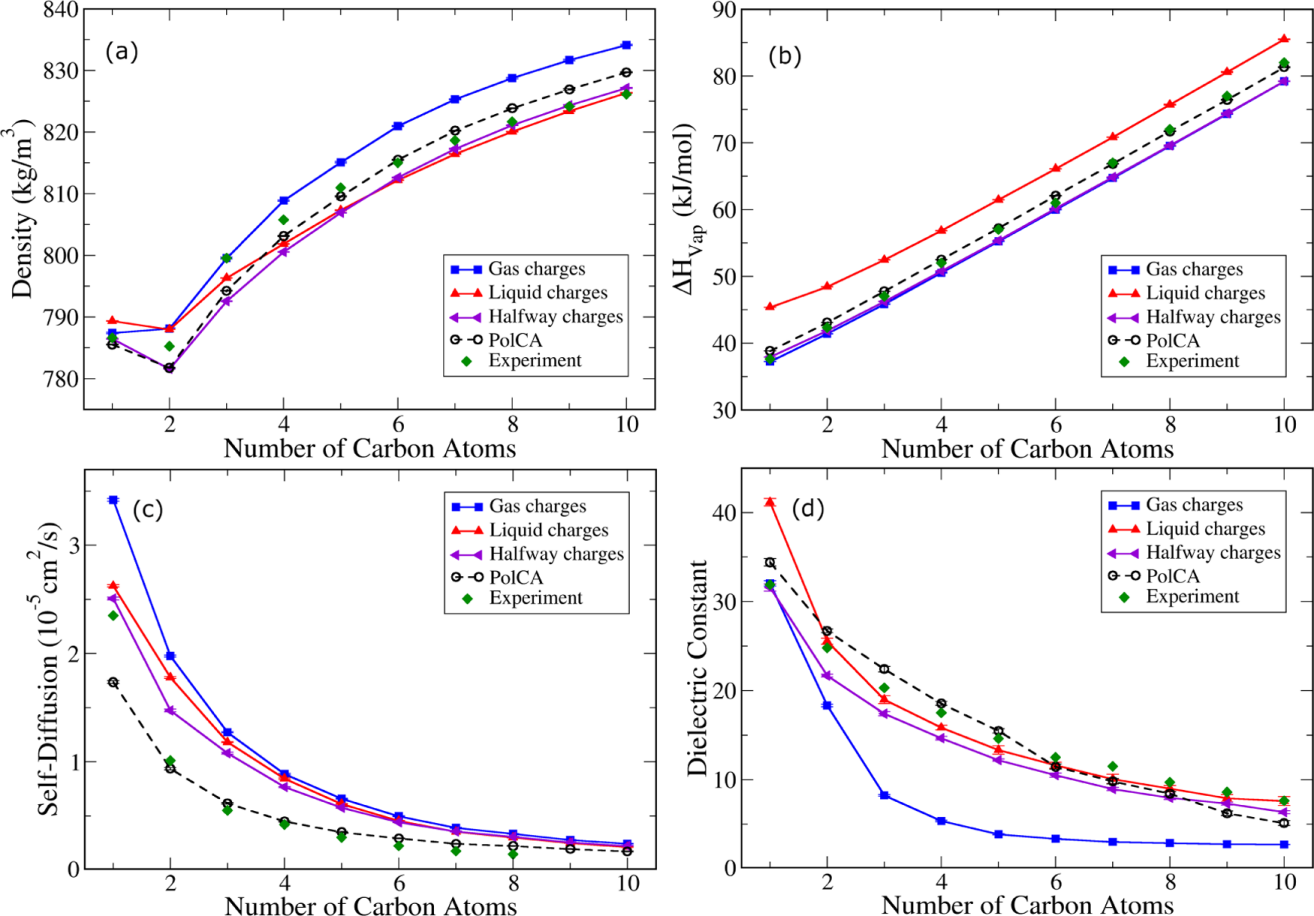
Predictions of the density (a), enthalpy of vaporization (b), self-diffusion coefficient (c), and dielectric constant (d) of linear alcohols up to 1-decanol, using models optimized from different sets of charges: gas-phase charges (blue squares), liquid-phase charges (red triangles), and halfway-polarized charges (purple triangles). The experimental data is plotted as green diamonds, while results from the previously optimized PolCA model^49^ are shown as black open circles.

As explained in Section 2, we have fitted LJ parameters against experimental data for methanol while keeping the point charges fixed using different charge assignment methods. We then tested how transferrable those parameters were to the entire series of linear alcohols up to 1-decanol. Figure 5 compares such predictions against experimental data for the density, enthalpy of vaporization, self-diffusion coefficient, and dielectric constant for three sets of point charges: (1) “gas-phase” charges, i.e., from a QM calculation carried out under vacuum; (2) “liquid-phase” charges, i.e., corresponding to the SCEE liquid-phase dipole moment of methanol; and (3) “halfway-polarized” charges, i.e., corresponding to the average dipole moment between the gas-phase and liquid-phase values. We also show the predictions of the PolCA model, empirically parameterized against experimental data for three alcohol molecules. The corresponding dipole moments and optimized LJ parameters are shown in Table 5.

**Table 5:** Force Field Optimization Results with Fixed Point Charges Representing Different Degrees of Polarization: Gas Phase, Liquid Phase, and “Halfway” Charges^a^

model	μ_M_	σ_O_	ε_O_	Δ*G*_Sol_
gas-phase charges	1.65	0.2495	1.04	–17.0 ± 0.1
liquid-phase charges	2.63	0.3312	1.83	–26.7 ± 0.1
halfway charges	2.13	0.2994	1.176	–19.8 ± 0.2
original PolCA^49^	2.07	0.2853	0.773	–20.7 ± 0.4

a For each optimized model, we report the model dipole moment (μ_M_ in Debye), the LJ parameters (σ in nm and ε in kJ/mol), and the predicted self-solvation free energy of methanol in kJ/mol (the experimental value is −20.5 kJ/mol).

The predictions of the three models for density and self-diffusion (Figure 5a and c) are all quite reasonable, although it is already apparent that the model with gas-phase charges performs somewhat worse than the other two. Conversely, the enthalpy of vaporization predictions (Figure 5b) with the gas-phase and halfway-charge models are quite good, but the model with liquid-phase charges significantly overestimates this property. The relatively poor performance of the gas-phase model for density and self-diffusion and of the liquid-phase model for enthalpy of vaporization is quite significant if we consider that they were fitted to match precisely those three properties for methanol. In this context, the comparison of the dielectric constant (Figure 5d) is particularly useful because it is a pure prediction—no dielectric constant data were used in training the models. As we can see, the gas-phase model performs rather poorly for this property, while the halfway-charge model performs somewhat better than the liquid-phase model and with accuracy comparable to that of the original PolCA model. In Table 5, we show the predictions of the self-solvation free energy of methanol for all of the models—again, this is a pure prediction since no solvation data were included in the parameterization process. For this property, the shortcomings of the gas-phase and liquid-phase models are even more evident since they significantly underestimate and overestimate, respectively, the magnitude of the solvation free energy of methanol. In contrast, the halfway-charge model yields predictions in very good agreement with the experimental data and quite close to the results of PolCA.

Although the LJ parameters (Table 5) are empirically fit to experimental data for methanol, they also shed some light on the reasons behind the performance of each model. Liquid-phase charges are too strongly polarized to provide an accurate representation of the PES of the liquid under a fixed-charge approximation. This over polarization is at least partially compensated by a very large LJ well-depth parameter—more than twice that of PolCA—that is necessary to increase the repulsion between neighboring hydroxyl groups and avoid the formation of too strong hydrogen bonds between them. The consequence is a poor balance between electrostatic and dispersion interactions, leading to the strong overestimation of the enthalpy of vaporization (Figure 5b) and the magnitude of the self-solvation free energy (Table 5). The gas-phase charges, on the other hand, are too weakly polarized to yield a reliable description of the PES of the liquid, and this under polarization cannot be completely compensated by tuning the LJ parameters of the model. In fact, the LJ diameter of oxygen for the gas-phase model is much lower than the van der Waals diameter of the oxygen atom (∼0.3 nm) due to the need to artificially strengthen the hydrogen bonds between neighboring hydroxyl groups. This leads to the observed overprediction of density since the excluded volume of each molecule decreases. On the other hand, the exceedingly low dipole moment leads to a strong underprediction of the dielectric constant, even after polarization corrections are applied.

In contrast to the gas-phase and liquid-phase models, when point charges are assigned so that they are halfway between the gas and liquid states, the predictions of the model are substantially improved. In fact, the model thus optimized performs only slightly worse than the original PolCA model for alcohols, where both the LJ parameters *and* the point charges were optimized to match the condensed-phase properties of several linear alcohols (more precisely, methanol, pentanol, and heptanol). The main reason for the worse performance of the halfway-charge model compared to PolCA is likely the difference in properties used for training. Using data for pentanol and heptanol in the parameterization leads to a better overall balance over the entire alcohol series, sometimes at the expense of a more precise fit for methanol. This can be clearly seen for the self-diffusion coefficient (Figure 5c) and, to a lesser extent, for the enthalpy of vaporization (Figure 5b). We recall that the aim of our analysis is not to obtain the best fixed-charge model for alcohols but to demonstrate that the halfway-charge concept leads to better predictions of bulk liquid properties than models with more extreme degrees of polarization.

**Figure 6:**
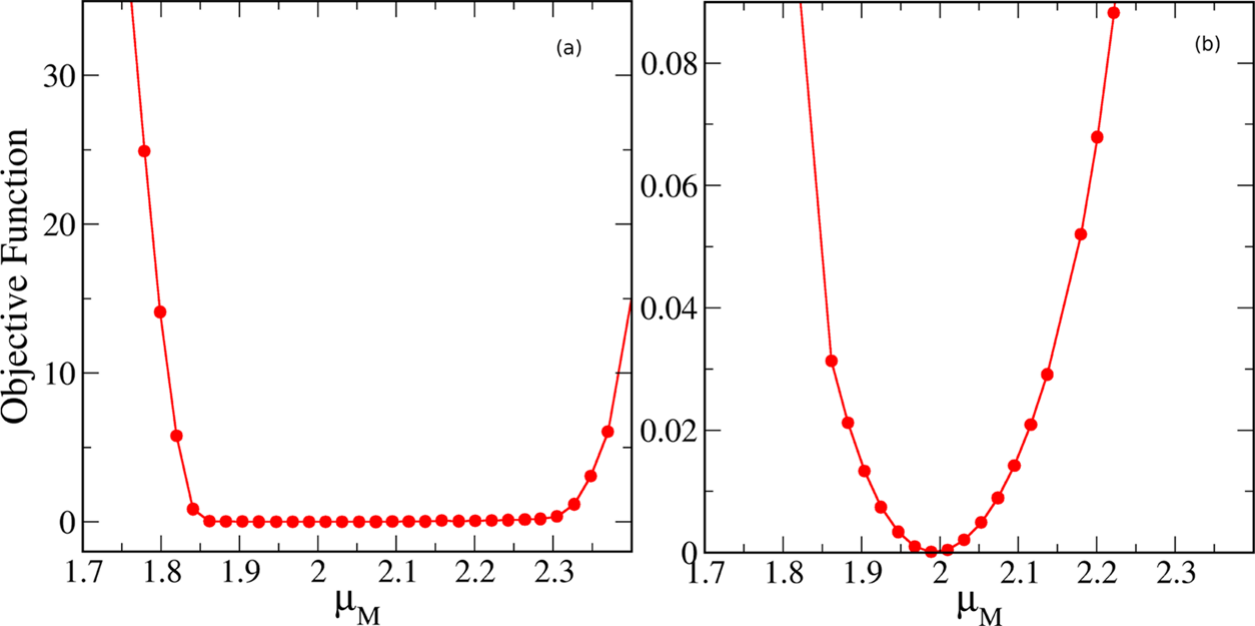
Value of the objective function of force field optimizations for methanol as a function of the dipole moment of the model over (a) the entire range of dipole moments and (b) over a narrow range close to the minimum.

We now take this analysis one step further and try to find out exactly what the optimal degree of polarization should be. For this purpose, we carried out a series of force field optimizations where the DDEC point charges were scaled to various levels between the gas and liquid dipole moments; for each of these charge sets, the LJ parameters were optimized to match the density, enthalpy of vaporization, and self-diffusion coefficient of methanol. In Figure 6, we plot the value of the objective function of the optimization against the dipole moment of the model (essentially determined by the value of the scaling factor applied to the original DDEC charges).

From Figure 6a, we can see that, in accordance with the above discussion, gas-phase and liquid-phase dipole moments (1.65 and 2.606 D, respectively, as calculated by the SCEE method) yield models that are too extreme in their degree of polarization and this cannot be fully compensated by simply adjusting the LJ parameters. Although there seems to be a rather wide range of dipole moments over which the model performs well, a close-up view (Figure 6b) reveals a clear minimum in the optimization at a model dipole moment value of 2.00 D. This is quite close to the average between the gas-phase and liquid-phase dipole moments of methanol (i.e., 2.127 D).

**Figure 7:**
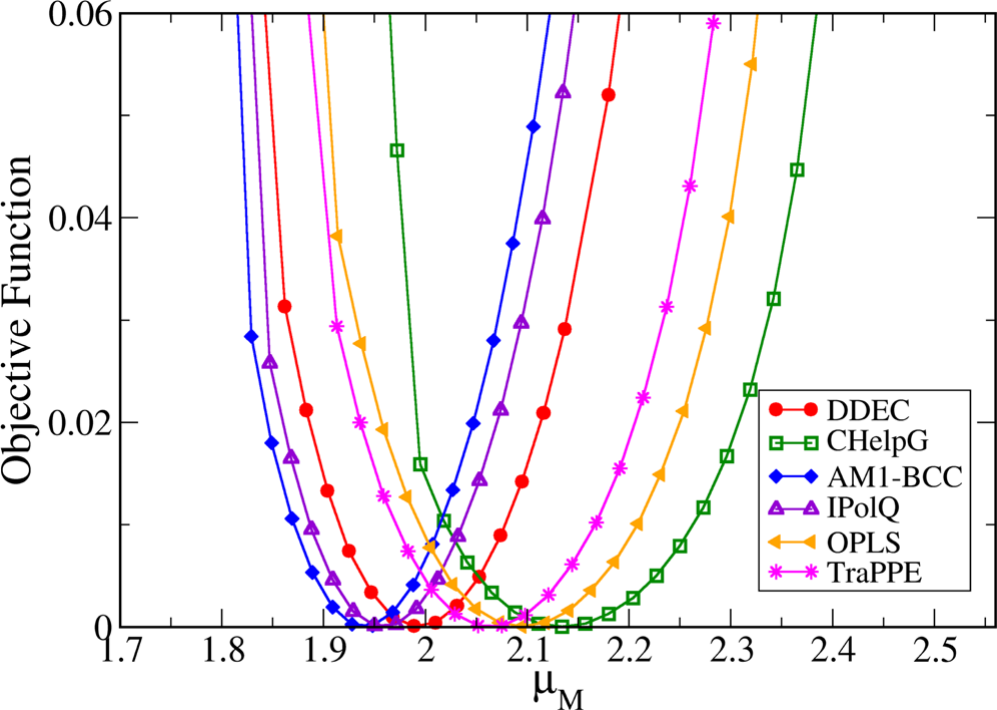
Value of the objective function of force field optimizations for methanol as a function of the dipole moment of the model over a narrow range close to the minimum for different initial charge sets (see Table 3).

In Figure 7, we show the same plot (only the close-up in the vicinity of the minimum) for several other sets of point charges, as described in Section 2.2 (see Table 3). As we can see, for all initial charge sets, a clear minimum is reached at values that are very close to the halfway point between the gas and the liquid dipole moments. As shown in Table 6, the optimum model dipole moments range from 1.94 to 2.14 D, compared to the “halfway” dipole moment of 2.127 D. Despite minor variations, the fact that point charge sets determined by significantly different methods (from QM calculations under vacuum or in a dielectric continuum, all the way to purely empirical charges) all converge to practically the same value for the optimal model dipole moment provides compelling evidence in support of the halfway-charge approach.

**Table 6:** Force Field Optimization Results Starting from Different Initial Point Charge Sets^a^

charge set	μ_M_	α
DDEC	2.00	0.96
CHelpG	2.14	0.95
AM1-BCC	1.94	1.01
IPolQ	1.96	0.97
OPLS	2.10	0.95
TraPPE	2.07	0.92

a For each optimized model, we report the model dipole moment (μ_M_ in Debye) and the scaling factor from the initial charge set (α).

Interestingly, the majority of the initial charge sets only require a small scaling to achieve the optimal degree of polarization—i.e., the values of *α* are all rather close to 1. One would expect this from the OPLS and TraPPE charge sets since they were empirically optimized to reproduce properties that depend on the PES of the fluid; as discussed above, the best performance for the model should be obtained when charges with an intermediate degree of polarization are employed, and that is indeed what we observe. A similar conclusion was drawn by Leontyev and Stuchebrukhov in their analysis of nonpolarizable water models.^45^ Our analysis also suggests that the IPolQ procedure, which employs the halfway-charge approach making use of a fully polarized reference liquid state,^25^ is able to yield charges with the correct degree of polarization for use in fixed-charge force fields without requiring further adjustments. A similar observation can be made for the AM1-BCC charge set, which, at least in the case of methanol, seems to yield charges with the appropriate intermediate degree of polarization. However, the AM1-BCC method was designed to reproduce charges obtained at the HF/6-31G* level of theory, which has been shown to lead to inconsistent results;^19^ hence, some caution is warranted before extrapolating this conclusion to different compounds.

Finally, it is perhaps more surprising to see that the DDEC and CHelpG charges, calculated from QM calculations in a PCM dielectric continuum, also yield the correct degree of polarization. This can be better understood in light of our recent studies of liquid-phase dipole moments of alcohols using the SCEE method.^29^ In that study, we showed that local effects, such as hydrogen-bond formation, account for just over half of the dipole enhancement in the liquid phase for methanol, the other half being due to mean-field effects. Since the PCM dielectric continuum model can only account for the latter, it yields a dipole moment that is nearly halfway polarized. Hence, the charges calculated using that method (in our case, the DDEC and CHelpG charge sets), perhaps fortuitously, lead to model dipole moments that are close to the average between the gas and liquid dipole moments. This observation, however, is unlikely to be generalizable to other molecules, where the relative importance of local and mean-field contributions to polarization are expected to be different from the ∼50/50 ratio observed for alcohols.

## 4 Conclusions

In this paper, we carried out a systematic assessment of two different approaches to implicitly account for polarization effects in classical nonpolarizable force fields—the QUBE force field,^38^ based on the “halfway-charge” approach,^1,25^ and the PolCA force field,^49^ based on the MDEC theory of Leontyev and Stuchebrukhov.^44^ Both approaches imply the use of point charges that represent a degree of polarization that is intermediate between the gas- and liquid-phase dipole moments. Such halfway-polarized charges provide the best possible representation of the potential energy surface of the liquid phase under the nonpolarizable approximation without the need to apply energetic polarization corrections to pure-component phase-change properties like enthalpy of vaporization or self-solvation free energy. However, a *post facto* polarization correction does need to be applied to accurately predict the bulk dielectric constant since it needs to reflect the Dipole Moment Surface of the real liquid.^47^ In the second part of the paper, we show that empirically optimized force fields can indeed yield more accurate predictions when the model dipole moments are intermediate between the gas and liquid degrees of polarization, supporting the use of halfway-polarized charges in a practical context. Furthermore, we have shown previously that models developed in such a way are more transferable to heterogeneous systems (e.g., solutions and mixtures), provided appropriate *post facto* polarization corrections are applied.^49^ Taken together, these conclusions provide a useful unified framework for future force field development and testing. However, one should always bear in mind that any nonpolarizable force field, even when employing the strategies discussed herein, is still not able to dynamically respond to changes in the polarization environment; for applications where this is an important factor, and the additional computational effort can be afforded, fully polarizable models are a better option.

A crucial element in the above framework is a realistic estimate of the real liquid-phase dipole moment of the molecule of interest, which accounts for both mean-field and local contributions to polarization. The recently proposed SCEE method^28,29^ is able to calculate accurate liquid-phase dipole moments in a computationally efficient way. In the first part of this paper, we showed that only when such a realistic liquid-phase dipole moment is used as a reference state does the QUBE force field yield internally consistent predictions of the enthalpy of vaporization. In contrast, the commonly used estimates based on dielectric continuum methods significantly underestimate the degree of polarization of the liquid since they do not account for local effects like hydrogen-bond formation and lead to inconsistent predictions. This implies that several recent approaches to account for polarization effects in generic nonpolarizable force fields are likely to be flawed because they consider a systematically under-polarized liquid as a reference state.

Several previous studies have tried to assess the performance of some of the new approaches to account for polarization effects on predicting solvation/hydration free energies with nonpolarizable models.^30,35−37,80,81^ The results were, at best, inconclusive—depending on the details of the QM level of theory and the charge determination method, charges polarized in a dielectric continuum sometimes led to improvements in predictions, but sometimes did not; the same was true for “halfway-polarized” charges determined by the IPolQ-Mod method. There are two main reasons for this state of affairs: (1) the LJ parameters of the force fields tested in the above comparisons (mainly GAFF) were determined in conjunction with HF/6-31G* charges and therefore are coupled to those charges. One should not expect, in general, that other charge models would perform particularly well without readjusting the LJ parameters at the same time and (2) the above comparisons assumed that the liquid-state polarization was adequately described by a dielectric continuum model, something we now know to be incorrect, at least for highly polar and hydrogen-bonding fluids. As discussed above, self-consistent and accurate predictions can only be expected if the correct reference state for the liquid is used—namely, an explicit solvent environment that can account for both mean-field and local contributions to polarization.

Our study was limited to only two compounds—water and methanol—due to the lack of reliable estimates of the real liquid-phase dipole moment for other classes of molecules. Although we believe the SCEE method is best suited for that purpose since it yields a good balance between accuracy and computational cost,^28,29^ it is still quite computationally intensive for routine use over thousands of different compounds. Therefore, a useful avenue for future research is to improve the SCEE approach to make it substantially faster while not compromising its accuracy. Once this is achieved, the calculation of reliable liquid-phase dipole moments can be incorporated into the force field parameterization workflow, with the ultimate goal of developing the next generation of nonpolarizable force fields that include a rigorous implicit account of polarization effects. Until that is achieved, however, a possible remedy is to use point charges and dipole moments obtained from QM calculations in a dielectric continuum as proxies for the halfway-polarized charges. For the particular cases of water and methanol, our results show that continuum dielectric model calculations yield dipole moments that are close to the halfway point. However, it is not yet clear to which extent this is fortuitous or indeed generalizable to a wider range of compounds. Studies to clarify this issue are currently underway.

## Data Availability

All data underpinning this publication are openly available from the University of Strathclyde KnowledgeBase at https://doi.org/10.15129/7e19e6b9-1bae-4928-bed8-5c8d29310c7c.

## Supporting Information Available

Bonded parameters for the QUBE force fields; additional details regarding the PolCA optimization procedure; equivalence between QUBE and halfway-charge approaches; demonstration of the quadratic dependence of the polarization energy components; and details about the Force Balance optimization of QUBE parameters (PDF)
