# Supplementary material for: What is the Optimal Dipole Moment for Nonpolarizable Models of Liquids?
Source: J Chem Theory Comput. 2023 Feb 24;19(6):1790–804. doi: 10.1021/acs.jctc.2c01123 (PMC10061682; doi:10.1021/acs.jctc.2c01123)
Supplement: Supplementary file 1 — ct2c01123_si_001.pdf [file ct2c01123_si_001.pdf]

Supporting Information for:

What is the Optimal Dipole Moment for Non-Polarizable Models of Liquids?

Miguel Jorge[1,], Maria Cecilia Barrera[2], Andrew W. Milne[1], Chris Ringrose[3], Daniel J. Cole[3]*

[1] Department of Chemical and Process Engineering, University of Strathclyde, 75 Montrose Street, Glasgow G1 1XJ, United Kingdom

[2] Strathclyde Institute of Pharmacy and Biomedical Sciences, University of Strathclyde, 161 Cathedral St, Glasgow G4 0RE, United Kingdom

[3] School of Natural and Environmental Sciences, Newcastle University, Newcastle upon Tyne NE1 7RU, United Kingdom

[*] Email: miguel.jorge@strath.ac.uk

S1. Bonded Parameters for QUBE Models

All of the bonded parameters were determined using the modified Seminario method [1], with values shown in Tables S1 and S2, and were kept constant in all of the QUBE models.

Table S1. *Bond stretching parameters for the QUBE models of water and methanol.*

**Bond type**	**Length (nm)**	**Force constant (kJ mol^{-1} nm^{-2})**
$O_W - H_W$	0.0957	235392
$C - O_H$	0.1413	112884
$C - H_C$	0.1094	133930
$O_H - H_O$	0.0956	235015

Table S2. *Angle bending parameters for the QUBE models of water and methanol.*

**Angle type**	**Angle (deg)**	**Force constant (kJ mol^{-1} rad^{-2})**
$H_W - O_W - H_W$	105.2	159.00
$O_H - C - H_C$	110.4	235.14
$H_C - C - H_C$	108.5	164.85
$C - O_H - H_O$	108.8	265.27

S2. Optimization Details for PolCA Models

For the scans of objective function vs model dipole moment (results shown in Figures 6 and 7 of the main paper), a learning set with 189 points (see Table S3 for parameter levels) was used to create meta-models that correlate the force fields' non-bonded parameters with the target properties: methanol's density, enthalpy of vaporisation and diffusion. Each property's meta-model was obtained by fitting the data to a second-order equation with cross-interaction terms [2]:

$$f(x_1,x_2,x_3,x_4) = \beta_0 + \sum_{i=1}^{4}\beta_i x_i + \sum_{i<j}\sum\beta_{ij}x_i x_j + \sum_{i=1}^{4}\beta_{ii}x_i^2 \quad (1)$$

where x_1,x_2,x_3 and x_4 were coded values of the hydroxyl group's partial charges, σ, and ε. The scans were carried out for several initial charge sets, shown in Table 3 of the main paper. In each case, the initial charges were scaled uniformly by a scaling factor to yield a series of model dipole moments between those of the gas- and liquid-phase dipoles. The LJ parameters were then optimized for each fixed value of the model dipole moment. In all cases, boundaries constraints were imposed in the optimisation to avoid obtaining LJ parameters that were far from the grid's parameter space, which might result in poor predictions. The boundaries were set to: σ from 0.27 nm to 0.31 nm and ε from 0.6 kJ/mol to 1.874 kJ/mol, and the meta-models were only allowed to marginally extrapolate outside those boundaries.

Table S3. *Levels of sigma (σ), epsilon (ε), oxygen's partial charge (q_O) and hydrogen's partial charge (q_H) used in the learning set for the model dipole moment scans.*

σ [nm]	0.278, 0.29, 0.294, 0.298 and 0.302
ε [kJ/mol]	0.7, 0.773, 0.846, 1.06, 1.274, 1.474, 1.674 and 1.874
q_O	-0.6, -0.65, -0.7, -0.75, -0.8 and -0.85
q_H	0.37, 0.402, 0.435, 0.468, 0.5 and 0.532

For the three models used to test the transferability of the parameters (i.e. results shown in Figure 5 of the main paper, corresponding to "gas charges", "liquid charges" and "halfway charges"), the meta-models were generated using the multivariate adaptive regression spline (MARS) [3,4] model. MARS is a weighted sum of basis functions, where each basis function is either a constant, a linear function, a hinge function, or a combination of these elements.

Hinge functions have the form $max\,(0,x-knot)$ or $max\,(0,knot-x)$, where $knot$ is a constant that divides the parameter's space of the input variable$x$. MARS is able to automatically determine the optimal number of basis functions and their parameters to prevent overfitting and maximise accuracy. Additionally, MARS allows the user to specify the maximum degree of interaction of each term, and in this work, we have chosen a value equal to 2. The learning set shown in Table S4, which consisted of a total of 225 points, was used to train the meta-models for the gas and halfway-charge models. For the liquid charge model, a larger grid (306 points), as shown in Table S5, was initially used to find an approximate location for the optimum, and then a refined meta-model was generated by only including points with σ values between 0.3 nm and 0.347 nm, since the optimum was within this region.

Table S4. *Levels of sigma (σ), epsilon (ε), oxygen's partial charge (q_O) and hydrogen's partial charge (q_H) used during development of the gas-phase and halfway-charge models.*

σ [nm]	0.278, 0.29, 0.294, 0.298, 0.302 and 0.326
ε [kJ/mol]	0.7, 0.773, 0.846, 1.06 and 1.274
q_O	-0.6, -0.7 and -0.8
q_H	0.37, 0.435 and 0.5

Table S5. *Levels of sigma (σ), epsilon (ε), oxygen's partial charge (q_O) and hydrogen's partial charge (q_H) used during development of the liquid-phase model.*

σ [nm]	0.278, 0.29, 0.294, 0.298, 0.302, 0.312, 0.326, 0.336 and 0.346
ε [kJ/mol]	0.7, 0.776, 0.846, 1.06, 1.274, 1.474, 1.674 and 1.874
q_O	-0.6, -0.65, -0.7, -0.75, -0.8 and -0.85
q_H	0.37, 0.402, 0.435, 0.468, 0.5 and 0.532

S3. Equivalence between QUBE and Halfway-charge approaches

The thermodynamic cycle depicted in Figure 1 of the main paper (and also in the Supporting Information of the original QUBE paper [5]) states that the total polarization correction (E_{Tot}) to be added to the results of molecular simulations can be written as a sum of a distortion (E_{Dist}) and a polarization (E_{Pol}) component:

$$E_{Tot} = E_{Dist}^{G \to M} + E_{Pol}^{M \to L} \tag{S.1}$$

where the superscripts on the terms of the right-hand side indicate that the distortion energy corresponds to a change from the unpolarized gas-phase (G) state to the intermediate model (M) state, while the polarization energy corresponds to a change from the model state to the fully polarized liquid (L) state. The QUBE approach further argues that the optimal degree of polarization of the model (and hence of the point charge values) is that which renders the total correction term to zero. In that case:

$$E_{Dist}^{G \to M} + E_{Pol}^{M \to L} = 0 \tag{S.2}$$

The polarization term can be further subdivided into an unfavorable distortion term and a favorable stabilization term (E_{Stab}) [6]:

$$E_{Pol}^{M \to L} = E_{Dist}^{M \to L} + E_{Stab}^{M \to L} \tag{S.3}$$

Under the linear response approximation, the magnitude of the stabilization energy is approximately twice that of the distortion energy but with opposite sign [6], i.e.:

$$E_{Stab}^{M \to L} \approx -2E_{Dist}^{M \to L} \tag{S.4}$$

therefore:

$$E_{Pol}^{M \to L} = E_{Dist}^{M \to L} - 2E_{Dist}^{M \to L} = -\, E_{Dist}^{M \to L} \tag{S.5}$$

Substituting in equation (S.2) yields:

$$E_{Dist}^{G \to M} = E_{Dist}^{M \to L} \tag{S.6}$$

In other words, at the optimal degree of polarization of the model, and under the linear response approximation, the distortion components of the first and third steps of the cycle shown in Figure 1 of the main paper have the same value.

To establish a correspondence with the halfway-charge approach, we use the simple expression derived by Berendsen et al. [7] for the distortion energy as a function of the change in dipole moment of the molecule:

$$E_{Dist}^{G \to M} = \frac{(\mu_M - \mu_G)^2}{2\alpha} \qquad \text{(S.7)}$$

where α is the molecular polarizability and μ is the dipole moment of the molecule at different states of polarization. Equation (S.7) was written for the process of polarizing the molecule from the gas to the model state, but can also be written for the model-to-liquid process:

$$E_{Dist}^{M \to L} = \frac{(\mu_L - \mu_M)^2}{2\alpha} \qquad \text{(S.8)}$$

Substituting equations (S.7) and (S.8) into equation (S.6) and manipulating, we obtain:

$$\frac{(\mu_M - \mu_G)^2}{2\alpha} = \frac{(\mu_L - \mu_M)^2}{2\alpha} \qquad \text{(S.9)}$$

$$(\mu_M - \mu_G)^2 = (\mu_L - \mu_M)^2$$

$$\mu_M - \mu_G = \mu_L - \mu_M$$

$$\mu_M = \frac{\mu_L + \mu_G}{2} \qquad \text{(S.10)}$$

In other words, the optimal degree of polarization found using the QUBE approach corresponds to a model dipole moment that is the average of the gas-phase and liquid-phase dipole moments, as prescribed by the halfway-charge approach.

S4. Quadratic dependence of the polarization energy components

As described in the main paper, in order to estimate the polarization energy contribution with the more realist reference state for the liquid phase dipole moment, obtained from an SCEE calculation [8,9], we make the assumption that the polarization energy depends quadratically on the difference between the actual and the reference dipole moment. This assumption is supported by several theoretical treatments of polarization.

Regarding the unfavorable distortion contribution (E_{Dist}), it has been shown [10,11] that, to leading order, this energy component is proportional to the difference between the dipole moments of the molecule in the model (μ_M) and in the ground state (i.e. in the unperturbed gas phase; μ_G), according to the well-known Berendsen expression [10]:

$$E_{Dist}^{G\to M} = \frac{(\mu_M - \mu_G)^2}{2\alpha} \quad \text{(S.7)}$$

This expression naturally neglects higher order terms [11]; however, it has been shown that the approximately quadratic dependence of the polarization energy on the dipole moment difference is retained even when quadrupole contributions are taken into account [8,12].

The polarization energy component (E_{Pol}), on the other hand, is comprised of two contributions, as described in the main paper: 1) the distortion energy due to polarizing the molecular wave function from the intermediate model state (μ_M) to the fully polarized liquid state (μ_L); 2) the favorable stabilization energy caused by the enhanced interaction between the polarized wave function and the surrounding liquid. The first of those contributions is given by the analogous of equation (S.7), but now written for the change between the model and the liquid dipole moments:

$$E_{Dist}^{M\to L} = \frac{(\mu_L - \mu_M)^2}{2\alpha} \quad \text{(S.8)}$$

A simple analytical expression for the stabilization energy (E_{Stab}) can be obtained from a simple Born model of solvation of a point dipole in a spherical cavity. This yields [13]:

$$E_{Stab} = -\left(\frac{\varepsilon - 1}{2\varepsilon + 1}\right)\frac{{\mu_L}^2}{R^3} \quad \text{(S.11)}$$

where ε is the static dielectric constant of the surrounding liquid, and R is the radius of the spherical cavity. This is, again, a very simplified treatment, but is used here simply to evidence the quadratic dependence of the stabilization energy on the liquid dipole moment.

As for the distortion component, it has been shown that this approximately quadratic dependence is maintained even when higher order terms are considered [8].

Combining the above two equations gives an expression for the polarization energy contribution:

$$E_{Pol} = E_{Dist}^{M \to L} + E_{Stab} = \frac{(\mu_L - \mu_M)^2}{2\alpha} - \left(\frac{\varepsilon - 1}{2\varepsilon + 1}\right)\frac{{\mu_L}^2}{R^3} \qquad \text{(S.12)}$$

The quadratic dependence on the dipole moment difference is further emphasised by the observation that the stabilization energy is found to be approximately equal to -2×E_{Dist} [14,15].

S5. Force field parameters for the QUBE water and methanol models after Force Balance optimization

For the halfway charge models, Force Balance [16] was used to optimize the methanol and water Lennard-Jones parameters against experimental liquid densities and heats of vaporisation target data. The objective function is calculated as a scaled sum of squared differences between calculated and reference data. We used scaling factors of 30 kg/m^3 and 3 kJ/mol for densities and heats of vaporization, respectively, to produce a dimensionless objective. Liquid simulation boxes containing 500 molecules were simulated under periodic boundary conditions in the NPT ensemble at the experimental reference temperature. A 1 fs time step was used, with liquid equilibration and production lengths of 0.2 ns and 3 ns, respectively. A Monte Carlo Barostat was used to maintain a constant pressure. An 8.5 Å non-bonded cut-off was used, along with a long-ranged dispersion correction and the particle mesh Ewald method for electrostatics. For heat of vaporisation calculations, gas phase simulations were performed with an infinite non-bonded cut-off.

Table S6 compares the original Lennard-Jones parameters for the QUBE water and methanol models against those fitted to the experimental density and enthalpy of vaporization using the Force Balance algorithm [16]. The point charges of the optimized model were kept fixed at the values that yield a model dipole moment that is halfway between that of the gas and of the pure liquid – i.e. using the halfway-charge approach – and are shown in Table S7.

Table S6. *Lennard-Jones parameters sigma (σ) and epsilon (ε) for water and methanol obtained with QUBE before and after Force Balance fitting. Atom types are: O for methanol oxygen, C for carbon, H_C for the aliphatic hydrogens, and O_W for water oxygen. Note that the LJ parameters of the (polar) hydroxyl and water hydrogen atoms were fixed at zero.*

	**Original**		**Optimized**	
**Atom type**	**σ (nm)**	**ε (kJ/mol)**	**σ (nm)**	**ε (kJ/mol)**
C	0.3220	0.2592	0.3228	0.261
H_C	0.2469	0.1505	0.2446	0.154
O	0.3159	0.5029	0.2960	0.502
O_W	0.3268	0.7301	0.3237	0.293

Table S7. *Point charges for water and methanol obtained with QUBE and adjusted to yield a dipole moment that is halfway between the gas and the liquid. Subscripts are: O for methanol oxygen, C for carbon, H_C for the aliphatic hydrogens, H_O for the hydroxyl hydrogen, O_W for water oxygen and H_W for water hydrogen atoms.*

**Atom Type**	C	H_C	O	H_O	O_W	H_W
**Charge**	0.1073	0.0333	-0.5914	0.3842	-0.8166	0.4083

References

[1] Allen, A. E.; Payne, M. C.; Cole, D. J. "Harmonic Force Constants for Molecular Mechanics Force Fields via Hessian Matrix Projection." *J. Chem. Theory Comput.* **2018**, *14*, 274-281.

[2] Khuri, A. I.; Mukhopadhyay, S. *Response surface methodology.* **2010**, *2*, 128−149.

[3] Friedman, J. H. "Multivariate adaptive regression splines." *The annals of statistics* 19, no. *1* (**1991**): 1-67.

[4] Friedman, J. H., Roosen, C. B. "An introduction to multivariate adaptive regression splines." *Statistical methods in medical research* 4, no. 3 (**1995**): 197-217.

[5] Cole, D. J.; Vilseck, J. Z.; Tirado-Rives, J.; Payne, M. C.; Jorgensen, W. L. "Biomolecular Force Field Parameterization via Atoms-in-Molecule Electron Density Partitioning" *J. Chem. Theory Comput.* **2016**, *12*, 2312−2323.

[6] Orozco, M.; Luque, F. J.; Habibollahzadeh, D.; Gao, J. "The polarization contribution to the free energy of hydration" *J. Chem. Phys.* **1995,** *102*, 6145-6152.

[7] Berendsen, H. J.; Grigera, J. R.; Straatsma, T. P. "The missing term in effective pair potentials" *J. Phys. Chem.* **1987**, *91*, 6269−6271.

[8] Jorge, M.; Gomes, J. R. B.; Milne, A. W. "Self-consistent electrostatic embedding for liquid phase polarization" *J. Mol. Liq.*, **2021**, *322*, 114550.

[9] Jorge, M.; Gomes, J. R. B.; Barrera, M. C. "The dipole moment of alcohols in the liquid phase and in solution" *J. Mol. Liq.*, **2022**, *356*, 119033.

[10] Berendsen, H. J.; Grigera, J. R.; Straatsma, T. P. "The missing term in effective pair potentials" *J. Phys. Chem.* **1987**, *91*, 6269−6271.

[11] Swope, W. C.; Horn, H. W.; Rice, J. E. "Accounting for polarization cost when using fixed charge force fields. I. Method for computing energy." *J. Phys. Chem. B* **2010**, *114*, 8621−8630.

[12] Milne, A. W.; Jorge, M. "Polarization Corrections and the Hydration Free Energy of Water" *J. Chem. Theory Comput.* **2019**, *15*, 1065–1078.

[13] Leontyev, I.; Stuchebrukhov, A. "Accounting for electronic polarization in non-polarizable force fields" *Phys. Chem. Chem. Phys.* **2011**, *13*, 2613-2626.

[14] Stone, A. *The Theory of Intermolecular Forces*; Clarendon Press: Oxford, **1996**.

[15] Orozco, M.; Luque, F. J.; Habibollahzadeh, D.; Gao, J. "The polarization contribution to the free energy of hydration" *J. Chem. Phys.* **1995,** *102*, 6145-6152.

[16] Wang, L.-P.; Chen, J.; Van Voorhis, T. "Systematic parametrization of polarizable force fields from quantum chemistry data." *J. Chem. Theory Comput.* **2013**, *9*, 452-460.
